# Refractoriness of aggressive behaviour to pharmacological treatment: cortical thickness analysis in autism spectrum disorder

**DOI:** 10.1192/bjo.2020.71

**Published:** 2020-08-07

**Authors:** Flavia Venetucci Gouveia, Jürgen Germann, Gabriel A. Devenyi, Rosa M. C. B. Morais, Ana Paula M. Santos, Erich T. Fonoff, Clement Hamani, Helena Brentani, M. Mallar Chakravarty, Raquel C. R. Martinez

**Affiliations:** Sunnybrook Research Institute, Canada; and Hospital Sirio-Libanes, Brazil; University Health Network; and CIC, Douglas Mental Health University Institute, McGill University, Canada; CIC, Douglas Mental Health University Institute, McGill University; and Department of Psychiatry, McGill University, Canada; Hospital Sirio-Libanes; and Department of Psychiatry, University of Sao Paulo, Medical School, Brazil; Department of Psychiatry, University of Sao Paulo, Medical School, Brazil; Department of Neurology, Division of Functional Neurosurgery of the Institute of Psychiatry, University of Sao Paulo, Medical School, Brazil; Sunnybrook Research Institute; Harquail Centre for Neuromodulation, Sunnybrook Health Sciences Centre; and Division of Neurosurgery, Sunnybrook Health Sciences Centre, University of Toronto, Canada; Department of Psychiatry, University of Sao Paulo, Medical School, Brazil; CIC, Douglas Mental Health University Institute, McGill University, Canada; Department of Psychiatry, McGill University, Canada; and Department of Biological and Biomedical Engineering, McGill University, Canada; Hospital Sirio-Libanes; and Department of Psychiatry, University of Sao Paulo, Medical School, Brazil

**Keywords:** Autistic spectrum disorders, intellectual disability, neuroimaging, cortical thickness, aggressive behaviour

## Abstract

Aggressive behaviour is a highly prevalent and devastating condition in autism spectrum disorder resulting in impoverished quality of life. Gold-standard therapies are ineffective in about 30% of patients leading to greater suffering. We investigated cortical thickness in individuals with autism spectrum disorder with pharmacological-treatment-refractory aggressive behaviour compared with those with non-refractory aggressive behaviour and observed a brain-wide pattern of local increased thickness in key areas related to emotional control and overall decreased cortical thickness in those with refractory aggressive behaviour, suggesting refractoriness could be related to specific morphological patterns. Elucidating the neurobiology of refractory aggressive behaviour is crucial to provide insights and potential avenues for new interventions.

Autism spectrum disorder (ASD) encompasses a range of neurodevelopmental disorders characterised by impairments in social interaction, communication deficits, restricted interests and repetitive behaviours.^1^ Although some individuals with ASD are able to live independently, others present with severe impairments causing great disability and impoverished quality of life (QoL).^1^ Furthermore, aggressive behaviours towards self (self-injurious behaviour) and others, is highly prevalent in individuals with ASD.^1,2^ Gold-standard aggressive behaviour therapies involve prescription drugs and psychotherapy and are effective in most patients.^1^ However, there is a subset (approximately 30%) that do not respond to treatment and are considered to have refractory aggressive behaviour (rAB).^2,3^ These patients are more challenging to treat and pose a high caregiver burden requiring specialised care. Given these challenges, efforts to find novel treatments, improve care and reduce suffering are paramount. Thus, studying the neurobiological underpinnings of rAB and identifying associated brain characteristics is essential to develop novel therapeutics.

It is believed that the neurocircuitry of aggressive behaviour involves cortical and limbic regions (for example prefrontal cortex; amygdala)^2,4,5^ and excessive aggressive behaviour occurs as a result of neurotransmission imbalances within these regions, but the neurobiological mechanisms underlying rAB are still unknown.^5^ Patients with aggressive behaviour are difficult to study as they are not collaborative in clinical settings and require deep sedation for image acquisition. However, as the chronic use of antipsychotics and benzodiazepines increases the risk of respiratory depression and death during sedation these types of interventions need to be restricted to the minimum.^2^ In this study, we had a unique opportunity to investigate the brain signature of individuals with rAB for a small group of patients with ASD with severe aggressive behaviour who had magnetic resonance imaging (MRI) for clinical purposes to investigate possible injuries to deep structures of the face, inner ear and head resulting from severe self-injury behaviour. Thus, we evaluated cortical thickness in individuals with rAB compared with patients with non-refractory aggressive behaviour (nrAB) to advance knowledge about the neurobiological mechanisms of rAB and provide insights into possible brain targets for neuromodulatory therapies.

## Method

### Participants

We report on ten patients with ASD associated with intellectual disabilities and severe aggressive behaviour, hallmarked by life-threatening self-injurious behaviour, aggression towards surroundings and others (rAB group: *n* = 3, 19–29 years; nrAB group: *n* = 7, 11–24 years; all males with normal karyotype 46, XY). rAB was defined as persistent aggressive behaviour despite previous trials of mono- and polypharmacy strategies of Food and Drug Administration-approved antipsychotics targeting aggressive behaviour^3^ (see Supplementary Table 1 available at https://doi.org/10.1192/bjo.2020.71: demographics and laboratory blood tests; Supplementary Table 2: medication history).

Questionnaires quantified severity of ASD (Childhood Autism Rating Scale, CARS), aggressive behaviour (Overt Aggression Scale), general motor agitation (Agitated Behaviour Scale) and QoL (Short Form Health Survey). Imaging acquisition was contemporaneous to surveys and laboratory blood tests. As patients presented with severe intellectual disability, neuropsychological evaluations were deferred and all questionnaires were reported by caregivers. The authors assert that all procedures contributing to this work comply with the ethical standards of the Research Ethical Board of the University of Sao Paulo, Medical School, Brazil and with the Helsinki Declaration of 1975, as revised in 2008. All procedures were approved by the REB (CAPPesq#742.331; CAAE#31828014.6.3001.5461) and written informed consent was obtained from the patient's caregivers.

### MRI acquisition and image processing

Scans were obtained under deep sedation on a 1.5 Tesla MRI system (Magnetom Espree, Siemens, Germany). *T*_1_-weighted structural images were acquired (slice thickness:1.0 mm, no gap, echo time (TE)/repetition time (TR): 5/300 ms, flip angle:45°, field of view (FOV): 240 mm, 1 × 0.9 × 0.9 mm or 1 × 0.5 × 0.5 mm voxel). Images were non-uniformity corrected using minc-bpipe-library (iterativeN4, https://github.com/CobraLab/minc-bpipe-library) and further processed using CIVET (v2.1.0; http://www.bic.mni.mcgill.ca/ServicesSoftware/CIVET) as described previously.^6^ Briefly, *T*_1_-weighted images were non-linearly registered to MNI152 space and tissue classification (grey matter, white matter, cerebrospinal fluid) was performed using tissue classification priors. The white matter and pial surfaces were extracted and registered to the MNI152 surface template. Cortical thickness was then computed at each vertex based on the distance between grey and white matter surfaces using the laplacian method and blurred with a 40 mm geodesic surface kernel.

### Statistical analysis

The RMINC (v.1.5.2.0, https://github.com/Mouse-Imaging-Centre/RMINC) package in R (versus3.4.4, https://www.r-project.org/) was used to perform cortical thickness analyses. To investigate brain-wide differences in cortical thickness patterns related to group (rAB versus nrAB), a linear model of thickness by group using individual mean cortical thickness as covariate to correct for global differences was computed at each vertex. All analyses were corrected for multiple comparisons using false discovery rate (FDR) at *P*_FDRcor_ < 0.05 threshold. Pearson correlations and *t*-tests were performed for demographics analysis.

## Results

**Fig. 1:**
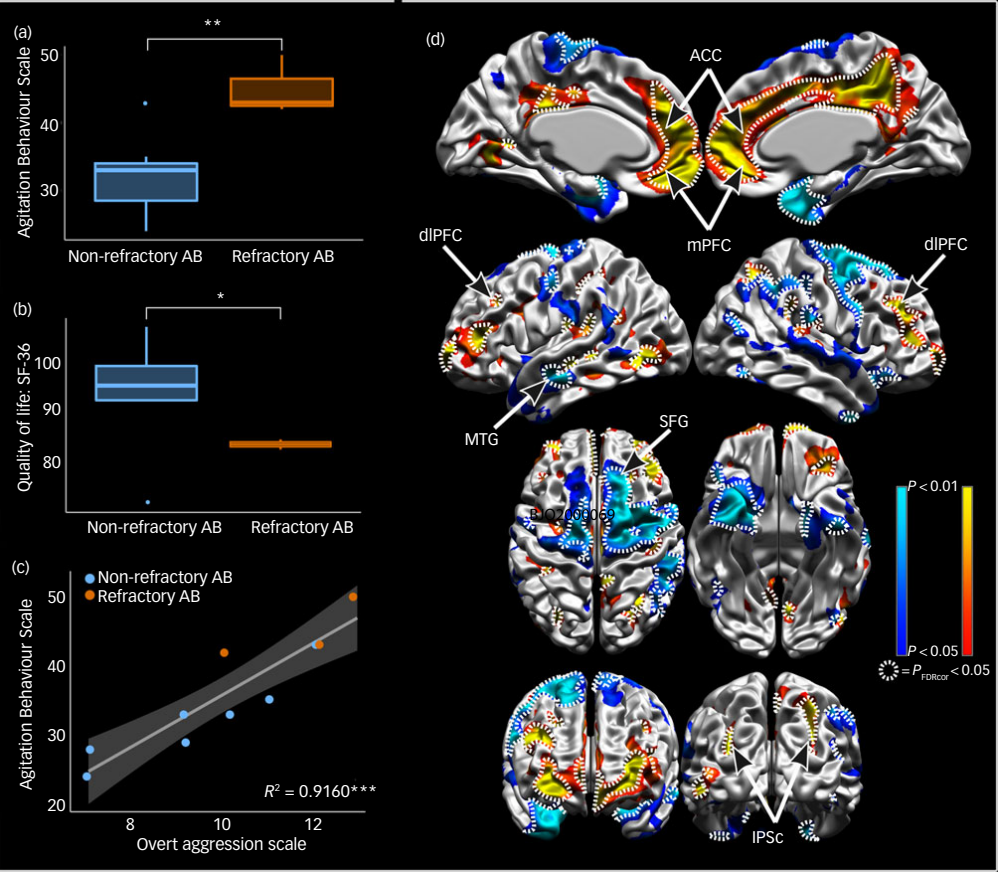
Results from questionnaires and the cortical thickness analysis. (a) Analysis of agitation using the Agitated Behaviour Scale. (b) Analysis of quality of life using the Short Form Health Survey (SF-36) questionnaire. (c) Correlation between agitation and aggressive behaviour. (d) Cortical thickness analysis using CIVET.

All participants in both groups had CARS score above 47 points indicating severe autism (rAB: mean 52 (s.d. = 3.6); nrAB: mean 51.6 (s.d. = 2.99). There were no differences between groups in age (rAB: mean 25 years (s.d. = 5.29); nrAB: mean 18.3 years (s.d. = 4.61); *t* = −1.9092, d.f. = 3.3922, *P* = 0.1415) and aggressive behaviour using the overt aggression scale (rAB: mean 11.7 (s.d. = 1.53); nrAB: mean 9.29 (s.d. = 1.89); *t* = −2.098, d.f. = 4.7965, *P* = 0.09235). The rAB group presented higher agitation (rAB: mean 45 (s.d. = 4.36); nrAB: mean 32.1 (s.d. = 6.07); *t* = −3.7763, d.f. = 5.448, *P* = 0.01103; Fig. 1(a)) and lower QoL (rAB: mean 83 (s.d. = 1); nrAB: mean 93.6 (s.d. = 11.4); *t* = 2.441, d.f. = 6.213, *P* = 0.04902; Fig. 1(a)) than the nrAB group. There was a positive correlation between agitation and aggressive behaviour (*t* = 6.4573, d.f. = 8, *P* = 0.0001968, *R*^2^ = 0.9159815; Fig. 1(c)).

Cold colours show areas where cortical thickness is diminished in refractory aggressive behaviour compared with non-refractory aggressive behaviour, warm colours show local cortical thickness increases. Dotted lines enclose significant areas (*P*_FDRcor_ < 0.05) after multiple comparison correction. Colours are thresholded at *P* < 0.05 uncorrected for multiple comparisons to show the extent and spatial pattern of the cortical thickness results. *P < 0.05; **P < 0.01; ***P < 0.001. AB, Aggressive behaviour; ACC, anterior cingulate cortex; dlPFC, dorsolateral prefrontal cortex; mPFC, medial prefrontal cortex; MTG, middle temporal gyrus; IPSc, intraparietal sulcus cortex; SFG, superior frontal gyrus.

The cortical thickness analysis showed that the rAB group have an overall cortical thickness reduction compared with the nrAB group (right hemisphere: *t* = −5.613, *P* = 0.0005; left hemisphere: *t* = −5.424, *P* = 0.0006). Vertex-wise relative cortical thickness analysis (correcting for mean cortical thickness), showed that local cortical thickness in the rAB group significantly increased (*P*_FDRcor_ < 0.05) bilateral in the dorsolateral prefrontal cortex (dlPFC), medial prefrontal cortex (mPFC), anterior cingulate cortex (ACC) and intraparietal sulcus cortex, and significantly decreased in the right superior frontal gyrus and left middle temporal gyrus (Fig. 1(d)).

## Discussion

The results suggest that individuals with rAB are more severely impaired than those with nrAB as they present with greater levels of agitation and lower QoL. The correlation between agitation and aggressive behaviour is an important factor when treating patients with aggressive behaviour as targeting this particular symptom could lead to reductions in aggressive behaviour and improvement in QoL. The cortical thickness analysis revealed significant differences in brain morphology between groups: (a) mean whole-brain cortical thickness is reduced in rAB and (b) brain-wide pattern shows local deviations in key regions related to emotional control and aggressive behaviour.^2,5^ These results suggest that refractoriness to treatment could be associated with a characteristic cortical thickness phenotype. It is important to highlight, however, that this analysis was performed in a small group of male patients with clinical indications for MRI. These patients presented with severe self-injury behaviour towards the face and head that could lead to fractures and brain injury. Only patients with normal radiological examination were included. Also, as some of the medications used for the control of aggressive behaviour are only prescribed in adulthood, the refractory group was composed of adults exclusively. All those in the refractory group had received at different times high-dose monotherapy and polypharmacy therapies. The possible side-effects of these therapies are poorly understood and worsening of the symptoms has been attributed to these treatments in some cases.^7,8^

It is known that individuals with ASD present with greater cortical thickness than healthy controls,^6^ however, the brain pattern differences found when the rAB and nrAB groups were compared were dissimilar to those found when comparing participants with ASD with healthy controls.^6^ The fine tuning within fronto-limbic-striatal circuits, in particular between the prefrontal cortex, amygdala and hypothalamus, is fundamental for behavioural control^5^ and aberrations affecting the structure or function of these regions can result in excessive aggressive behaviour.^5^

Non-invasive neuromodulation therapies (for example transcranial magnetic stimulation; transcranial direct current stimulation) are currently being investigated for the treatment of neuropsychological symptoms, such as aggressive behaviour and agitation.^9^ These techniques can alter cortical excitability, resulting in widespread connectivity modifications and restoration of normal connectivity patterns.^10,11^ More recently, low-intensity focused ultrasound has emerged as a neuromodulatory alternative to target deep cortical areas and subcortical structures.^12^ With this rationale in mind, it is possible to propose the use of neuromodulatory therapies targeting one or more of these key brain regions (for example dlPFC, mPFC, ACC) to modulate local neural activity in an attempt to restore medication responsiveness in rAB. Further work with this group with rare and severe conditions is necessary to better understand the causes of cortical thickness changes, improve treatment options and reduce suffering.

## Data Availability

All data generated and analysed during this study are available from the corresponding author upon reasonable request.

## Supplementary material

For supplementary material accompanying this paper visit http://dx.doi.org/10.1192/bjo.2020.71. click here to view supplementary material
