# Refractoriness of aggressive behaviour to pharmacological treatment: cortical thickness analysis in autism spectrum disorder – ERRATUM

**DOI:** 10.1192/bjo.2020.91

**Published:** 2020-08-27

**Authors:** Flavia Venetucci Gouveia, Jürgen Germann, Gabriel A. Devenyi, Rosa M. C. B. Morais, Ana Paula M. Santos, Erich T. Fonoff, Clement Hamani, Helena Brentani, M. Mallar Chakravarty, Raquel C. R. Martinez

**Keywords:** Autistic spectrum disorders, intellectual disability, neuroimaging, cortical thickness, aggressive behaviour, erratum

This article fails to state the coequal contribution to the article of its first two authors, Flavia Venetucci Gouveia and Jürgen Germann. This note was deleted accidentally during the production process, for which the publisher apologises.
