# Supplementary material for: Refractoriness of aggressive behaviour to pharmacological treatment: cortical thickness analysis in autism spectrum disorder
Source: BJPsych Open. 2020 Aug 7;6(5):e85. doi: 10.1192/bjo.2020.71 (PMC7453794; doi:10.1192/bjo.2020.71)
Supplement: Supplementary file 1 [file bjosup.zip › S205647242000071Xsup002.zip!/Supplementary_Table2.docx]

| Supplementary Table 2. Medication history. | | |
| --- | --- | --- |
| **Case** | **Group** | **Medication History** |
| 01 | Refractory-AB | Aripiprazole; Biperiden; Carbamazepine; Chlorpromazine; Clozapine; Divalproex sodium; Haloperidol; Levomepromazine; Olanzapine; Promethazine; Quetiapine fumarate; Risperidone; Thioridazine; Ziprasidone |
| 02 | Refractory-AB | Aripiprazole; Benzodiazepines; Biperiden; Carbamazepine; Chlorpromazine; Clonidine; Clozapine; Divalproex sodium; Haloperidol; Levomepromazine; Olanzapine; Promethazine; Risperidone; Thioridazine; Topiramate |
| 03 | Refractory-AB | Aripiprazole; Biperiden; Carbamazepine; Chlorpromazine; Clomipramine; Clonazepam; Divalproex sodium; Haloperidol; Levomepromazine; Olanzapine; Oxcarbazepine; Promethazine; Quetiapine fumarate; Risperidone; Sertraline; Thioridazine; Topiramate; Valproate; Ziprasidone |
| 04 | Non-Refractory-AB | Clobazam; Levomepromazine; Lithium; Risperidone; Topiramate; Valproate |
| 05 | Non-Refractory-AB | Valproate; Carbamazepine; Lithium; Promethazine; Quetiapine fumarate; Risperidone |
| 06 | Non-Refractory-AB | Clobazam; Clonidine; Clozapine; Lamotrigine |
| 07 | Non-Refractory-AB | Biperiden; Haloperidol; Valproate |
| 08 | Non-Refractory-AB | Biperiden; Levomepromazine; Lithium; Olanzapine; Valproate |
| 09 | Non-Refractory-AB | Carbamazepine; Haloperidol; Levomepromazine |
| 10 | Non-Refractory-AB | Levomepromazine; Nitrazepam; Phenobarbital; Risperidone |
| Abbreviation: AB: Aggressive Behaviour | | |
