# Supplementary material for: Refractoriness of aggressive behaviour to pharmacological treatment: cortical thickness analysis in autism spectrum disorder
Source: BJPsych Open. 2020 Aug 7;6(5):e85. doi: 10.1192/bjo.2020.71 (PMC7453794; doi:10.1192/bjo.2020.71)
Supplement: Supplementary file 1 [file bjosup.zip › S205647242000071Xsup002.zip!/Supplementary_Table1.docx]

| Supplementary Table 1. Demographics and laboratory blood tests. | | | | | | | | | | |
| --- | --- | --- | --- | --- | --- | --- | --- | --- | --- | --- |
| **Demographics** | *Refractory Aggressive Behaviour Group* | | | *Non-refractory Aggressive Behaviour Group* | | | | | | |
|  | **c01** | **c02** | **c03** | **c04** | **c05** | **c06** | **c07** | **c08** | **c09** | **c10** |
| Gender | M | M | M | M | M | M | M | M | M | M |
| Age | 19 | 27 | 29 | 20 | 21 | 19 | 11 | 24 | 13 | 20 |
| CARS | 55 | 53 | 48 | 54 | 47 | 51 | 48 | 54 | 53 | 54 |
| Total OAS | 13 | 10 | 12 | 10 | 9 | 11 | 7 | 12 | 9 | 7 |
| Subscale OAS: Verbal | 1 | 1 | 1 | 1 | 1 | 0 | 1 | 1 | 1 | 1 |
| Subscale OAS: Self | 4 | 1 | 4 | 3 | 2 | 3 | 0 | 4 | 1 | 3 |
| Subscale OAS: Objects | 4 | 4 | 3 | 3 | 3 | 4 | 4 | 4 | 3 | 1 |
| Subscale OAS: Others | 4 | 4 | 4 | 3 | 2 | 4 | 2 | 3 | 4 | 2 |
| ABS | 50 | 42 | 43 | 33 | 33 | 35 | 24 | 43 | 29 | 28 |
| SF-36 | 84 | 83 | 82 | 96 | 92 | 71 | 107 | 102 | 95 | 92 |
| **Blood Tests** |  | | | | | | | | | |
| Oxytocin (pg/mL) | 40.46 | 205.97 | 86.82 | 106.40 | 39.88 | 90.68 | 73.97 | 133.91 | 84.87 | 513.23 |
| Serotonin (pg/mL) | 92.42 | 617.01 | 0.97 | 491.54 | 57.75 | 180.1 | 328.64 | 71.53 | 40.93 | 0.97 |
| Vasopressin (pg/mL) | 8.87 | 7.06 | 4.53 | 10.54 | 4.53 | 10.45 | 7.06 | 14.38 | 4.53 | 10.97 |
| TSH (mIU/L) | 1.83 | 0.89 | 1.75 | 2.95 | 1.84 | 2.09 | 6.93 | 4.59 | 3.26 | 2.91 |
| T4 (ug/dL) | 7.50 | 10.10 | 3.80 | 7.10 | 4.38 | 5.40 | 6.70 | 7.40 | 6.30 | 9.60 |
| Free T4 (ng/dL) | 1.58 | 1.44 | 1.16 | 1.11 | 1.19 | 1.51 | 1.42 | 1.15 | 1.42 | 1.00 |
| T3 (mg/dL) | 0.13 | 0.11 | 0.08 | 0.13 | 0.11 | 0.09 | 0.14 | 0.12 | 0.11 | 0.12 |
| LH (IU/L) | 1.70 | 3.40 | 1.80 | 2.10 | 4.90 | 3.60 | 1.20 | 5.80 | 1.20 | 3.80 |
| FSH (IU/L) | 1.30 | 5.90 | 2.00 | 7.60 | 3.00 | 5.30 | 1.00 | 1.40 | 2.30 | 1.50 |
| Estradiol (pg/mL) | 17.00 | 55.00 | 30.00 | 15.00 | 23.07 | 15.00 | 15.00 | 15.00 | 21.10 | 36.00 |
| Prolactin (mg/dL) | 0.57 | 3.20 | 60.10 | 70.50 | 44.90 | 14.80 | 47.50 | 46.10 | 1.30 | 3.70 |
| Progesterone (ng/mL) | 0.30 | 0.30 | 0.30 | 0.80 | 0.60 | 0.80 | 0.50 | 0.30 | 0.80 | 0.70 |
| Total Testosterone (ng/mL) | 336.0 | 412.0 | 409.0 | 249.0 | 316.0 | 346.0 | 88.0 | 473.0 | 261.0 | 514.0 |
| Free Testosterone (pmol/L) | 169.0 | 535.0 | 407.0 | 164.0 | 223.0 | 225.0 | 36.0 | 327.0 | 159.0 | 455.0 |
| SHBG (nmol/L) | 57.00 | 17.00 | 26.00 | 34.00 | 31.60 | 36.80 | 62.60 | 35.60 | 39.00 | 23.10 |
| Cortisol (mg/dL) | 4.60 | 1.90 | 5.90 | 10.80 | 17.10 | 18.20 | 8.50 | 10.70 | 21.50 | 5.00 |
| ACTH (pg/mL) | 12.10 | 10.20 | 22.90 | 60.80 | 40.50 | 37.90 | 39.10 | 36.50 | 78.70 | 19.00 |
| Sodium (mEq/L) | 144 | 143 | 92 | 144 | 140 | 128 | 141 | 141 | 142 | 142 |
| Potassium (mEq/L) | 4.30 | 4.50 | 4.60 | 4.90 | 4.50 | 4.30 | 4.50 | 4.60 | 4.00 | 4.70 |
| Calcium (mg/dL) | 9.50 | 8.40 | 9.20 | 10.10 | 9.60 | 9.30 | 9.60 | 10.00 | 10.10 | 9.70 |
| Magnesium (mg/dL) | 1.92 | 1.83 | 1.71 | 2.15 | 2.07 | 1.75 | 1.91 | 2.14 | 1.89 | 2.18 |
| Urea (mg/dL) | 16.00 | 39.00 | 16.00 | 22.00 | 16.00 | 14.00 | 37.00 | 16.00 | 25.00 | 16.00 |
| Creatinine (mg/dL) | 0.82 | 0.96 | 0.73 | 0.64 | 0.90 | 0.53 | 0.70 | 0.91 | 0.84 | 0.67 |
| Amylase (U/L) | 39.00 | 50.00 | 55.00 | 26.00 | 52.00 | 56.00 | 72.00 | 70.00 | 69.00 | 35.00 |
| Lipase (U/L) | 21.00 | 28.00 | 29.00 | 22.00 | 24.00 | 13.00 | 26.00 | 45.00 | 24.00 | 15.00 |
| Total Bilirubin (mg/dL) | 0.37 | 0.54 | 0.31 | 0.50 | 0.18 | 0.26 | 0.33 | 0.42 | 0.52 | 0.29 |
| Direct Bilirubin (mg/dL) | 0.20 | 0.26 | 0.14 | 0.25 | 0.08 | 0.14 | 0.17 | 0.19 | 0.21 | 0.04 |
| Indirect Bilirubin (mg/dL) | 0.17 | 0.28 | 0.17 | 0.25 | 0.10 | 0.12 | 0.16 | 0.23 | 0.31 | 0.25 |
| AST (U/L) | 22.00 | 13.00 | 21.00 | 18.00 | 23.00 | 13.00 | 19.00 | 23.00 | 14.00 | 25.00 |
| ALT (U/L) | 22.00 | 14.00 | 13.00 | 19.00 | 21.00 | 25.00 | 8.00 | 20.00 | 10.00 | 14.00 |
| GGT (U/L/) | 48.00 | 15.00 | 87.00 | 29.00 | 131.0 | 51.00 | 32.00 | 26.00 | 16.00 | 23.00 |
| Total Cholesterol (mg/dL) | 247.0 | 147.0 | 204.0 | 152.0 | 163.0 | 89.0 | 134.0 | 166.0 | 135.0 | 131.0 |
| Triglycerides (mg/dL) | 173.0 | 112.0 | 150.0 | 92.0 | 215.0 | 45.0 | 105.0 | 110.0 | 109.0 | 82.0 |
| HDL (mg/dL) | 70.00 | 34.00 | 49.00 | 56.00 | 34.00 | 38.00 | 51.00 | 51.00 | 48.00 | 24.00 |
| LDL (mg/dL) | 128.0 | 92.0 | 125.0 | 78.0 | 86.0 | 38.0 | 64.0 | 94.0 | 68.0 | 90.0 |
| VLDL (mg/dL) | 29.00 | 21.00 | 23.00 | 22.00 | 30.00 | 13.00 | 19.00 | 21.00 | 19.00 | 17.00 |
| Glucose (mg/dL) | 96.0 | 64.0 | 79.0 | 87.0 | 96.0 | 71.0 | 78.0 | 65.0 | 103.0 | 89.0 |
| Glycated Hemoglobin (%) | 5.2 | 4.4 | 4.7 | 5.4 | 5.5 | 5.2 | 5.5 | 4.6 | 5.0 | 4.6 |
| Platelets (th/mm3) | 158.0 | 127.0 | 161.0 | 102.0 | 269.0 | 267.0 | 277.0 | 262.0 | 189.0 | 163.0 |
| MPV (fL) | 9.00 | 11.50 | 9.30 | 13.20 | 11.10 | 10.00 | 10.10 | 11.50 | 10.20 | 10.50 |
| Erythrocytes (mil/mm3) | 4.70 | 5.31 | 4.43 | 4.62 | 4.78 | 4.53 | 5.33 | 4.60 | 5.40 | 5.12 |
| Hemoglobin (g/dL) | 14.40 | 15.00 | 13.90 | 13.50 | 14.30 | 13.10 | 14.50 | 13.60 | 16.30 | 15.50 |
| Hematocrit (%) | 42.9 | 45.1 | 39.5 | 40.5 | 40.6 | 36.4 | 43.6 | 41.8 | 46.9 | 46.2 |
| MCV (fL) | 91.30 | 84.90 | 89.20 | 87.70 | 97.40 | 80.40 | 81.80 | 90.90 | 86.90 | 90.20 |
| MCH (pg) | 30.60 | 28.20 | 31.40 | 29.20 | 34.30 | 28.90 | 27.20 | 26.60 | 30.30 | 30.30 |
| MCHC (g/dL) | 33.60 | 33.30 | 35.20 | 33.30 | 35.20 | 36.00 | 33.30 | 32.50 | 34.80 | 33.50 |
| RDW-CV (%) | 13.4 | 14.3 | 12.9 | 13.9 | 12.9 | 13.9 | 13.6 | 12.5 | 13.0 | 13.1 |
| RDW-SD (fL) | 44.60 | 44.50 | 42.30 | 45.10 | 43.40 | 40.60 | 40.80 | 41.40 | 41.20 | 43.40 |
| Leukocytes (th/mm3) | 5.45 | 5.46 | 4.34 | 3.69 | 5.53 | 6.26 | 6.02 | 6.83 | 6.27 | 5.92 |
| Neutrophils (mil/mm3) | 1.71 | 3.84 | 1.54 | 1.23 | 2.86 | 2.74 | 2.34 | 4.12 | 2.93 | 4.71 |
| Eosinophils (mil/mm3) | 0.56 | 0.13 | 0.02 | 0.22 | 0.17 | 0.35 | 0.04 | 0.08 | 0.03 | 0.02 |
| Basophils (mil/mm3) | 0.01 | 0.04 | 0.01 | 0.01 | 0.02 | 0.03 | 0.01 | 0.04 | 0.02 | 0.02 |
| Lymphocyte (mil/mm3) | 2.44 | 1.05 | 2.07 | 1.85 | 1.58 | 1.55 | 3.14 | 2.21 | 2.64 | 0.70 |
| Monocytes (mil/mm3) | 0.73 | 0.40 | 0.70 | 0.38 | 0.38 | 1.59 | 0.49 | 0.38 | 0.65 | 0.47 |
| **Abbreviations: ABS:** Agitated Behaviour Scale; **ALT:** Alanine Aminotransferase; **AST:** Aspartate Aminotransferase; **ACTH:** Adrenocorticotropic Hormone; **CARS**: Childhood Autism Rating Scale; **Free T4:** Free Thyroxine; **FSH:** Follicle-Stimulating Hormone; **GGT:** Gamma Glutamyl Transpeptidase; **HDL:** High-Density Lipoprotein; **LDL:** Low-Density Lipoprotein; **LH:** Luteinizing Hormone; **MCH:** Mean Cell Hemoglobin; **MCHC:** Mean Cell Hemoglobin Concentration; **MCV:** Mean Corpuscular Volume; **MPV:** Mean Platelet Volume; **RDW-CV:** Red Cell Distribution Width – Cell Volume; **RDW-SD:** Red Cell Distribution Width – Standard Deviation; **SF-36:** Short Form Health Survey; **SHBG:** Sex Hormone Binding Globulin; **TSH:** Thyroid-Stimulating Hormone; **T3:** Triiodothyronine; **T4:** Thyroxine; **VLDL:** Very Low Density Lipoprotein Cholesterol. | | | | | | | | | | |
